# Using SPOT–5 HRG Data in Panchromatic Mode for Operational Detection of Small Ships in Tropical Area

**DOI:** 10.3390/s8052959

**Published:** 2008-05-06

**Authors:** Christina Corbane, Fabrice Marre, Michel Petit

**Affiliations:** 1 ESPACE Unit, Institut de Recherche pour le Développement, Maison de la télédétection, 500 rue JF Breton, F34093 Montpellier cedex 5; E–mails: christina.corbane@ird.fr; michel.petit@ird.fr; 2 ESPACE Unit, Institut de Recherche pour le Développement, Centre IRD de Cayenne, Route de Montabo, PO Box 165, F97323, Cayenne, Guyane Française

**Keywords:** Automatic ship detection, SPOT-5 HRG data, high spatial resolution, Neural Networks, Genetic Algorithm, maritime surveillance

## Abstract

Nowadays, there is a growing interest in applications of space remote sensing systems for maritime surveillance which includes among others traffic surveillance, maritime security, illegal fisheries survey, oil discharge and sea pollution monitoring. Within the framework of several French and European projects, an algorithm for automatic ship detection from SPOT–5 HRG data was developed to complement existing fishery control measures, in particular the Vessel Monitoring System. The algorithm focused on feature–based analysis of satellite imagery. Genetic algorithms and Neural Networks were used to deal with the feature–borne information. Based on the described approach, a first prototype was designed to classify small targets such as shrimp boats and tested on panchromatic SPOT–5, 5–m resolution product taking into account the environmental and fishing context. The ability to detect shrimp boats with satisfactory detection rates is an indicator of the robustness of the algorithm. Still, the benchmark revealed problems related to increased false alarm rates on particular types of images with a high percentage of cloud cover and a sea cluttered background.

## 1. Introduction

Ship detection is a key requirement for monitoring traffic, fisheries and for associating ships with oil discharges. Provision of a well designed maritime surveillance and control system capable of tracking ships is therefore essential and would be a vital interest to a variety of users ranging from local authorities to defence organizations, national and international. The Vessel Monitoring System (VMS) that relies on a ship–born component provides the authorities with a continuous monitoring of vessels' location and movements in real time. However, many ships are not equipped with these systems, for example smaller fishery vessels and passenger boats do not have to apply with the existing directives (e.g. EC directive 2002/59/EC). One has to resort to remote sensing using Earth Observation (EO) satellites in order to obtain information on these vessels. In that sense, remote sensing is regarded as a technology to support the active system with passive measurements for non–cooperating ships, sensing of non–harbour regions and monitoring purposes [1]. Space–based imaging for ship detection and maritime traffic surveillance has often formed part of major research efforts in the fields of automatic target detection and recognition. Ship detection with satellite based on Synthetic Aperture Radar (SAR) was first demonstrated by the experimental SEASAT in 1978. With later first–generation satellites such as ERS–1, JERS–1, ERS–2 the field has reached some maturity [2]. With the advent of the second generation of radar satellites such as ENVISAT and RADARSAT–1, ship detection capabilities were once and for all established thanks to advanced specific processing taking advantage of the huge amount of information that can be retrieved from low level products [3]. The third generation satellites TerraSAR–X, Cosmo–Skymed (CSK), RADARSAT–2, ALOS are somewhat different, as the design is more heavily influenced by the requirement for high–resolution imagery on land. Some of this will lead to improved detection and classification functionality of ship targets. However, in general, the increase in this additional information will go hand in hand with a decrease in swath width introducing some limitations regarding maritime use [4].

SAR has been studied extensively and most recently reviewed in [5]. Even though SAR imagery is advantageous due to its ability to scan large areas and its independence from cloud and light conditions, individual identification and classification of vessels at a higher detail level remains a difficult task. Besides, due to the presence of speckle and the reduced dimensions of the targets compared with the sensor spatial resolution, the automatic interpretation of SAR images is often complex even though vessels undetected are sometimes visible by eye [6]. Compared to the large amount of investigations on the feasibility of satellite–based SAR for ship detection purposes, far less research and development activity has taken place in automatic detection and classification of vessels using optical imagery than using SAR imagery [7]. This is a consequence of the novelty of the high resolution optical satellite sensors, the problem of clouds, and the fact that the swath of high resolution imagery is relatively small, making it less suitable for surveillance over the oceans. However, high spatial resolution can complement SAR since it is most suitable for ship classification and it permits the detection of wooden and fibreglass boats, which are difficult to detect with radar [8]. In this context, there is an imperative need for a system that automatically detects ship patterns from high spatial resolution imagery in an operational framework.

This paper proposes an approach for the detection and classification of ships from high spatial resolution optical imagery. It was developed in the framework of French and European projects (*IBIS*, *DECLIMS* and *LIMES*) to complement existing fishery control measures, in particular the VMS. The algorithm focuses on feature–based analysis of satellite imagery. Genetic algorithms (GAs) and neural networks (NN) are used to deal with the feature–borne information. Based on the described approach, a first prototype was designed to classify small targets such as shrimp boats and tested on 5–m resolution panchromatic SPOT5–HRG data.

## 2. Methodology

### 2.1. Method Overview

The algorithm is a three–step object detection task consisting of the following stages:

– segmentation or predetection of ship patterns,
– feature extraction,
– classification.

The segmentation aims at detecting potential ship targets. It involves a pre–processing stage for the purpose of removing noise. Then, image objects corresponding to potential ship targets are created by means of a region–growing algorithm.

During the feature extraction stage, image objects are characterized by spectral, shape and textural features. The feature extraction step is concerned with finding transformations to map features to a lower dimensional space for enhanced class separability and optimized performance. Because of their robustness, GAs are considered a suitable tool to address the optimization problem [9]. The GA–driven selection procedure provides a vector of feature values corresponding to a series of feature combinations that is passed to the subsequent classification stage.

**Figure 1.**
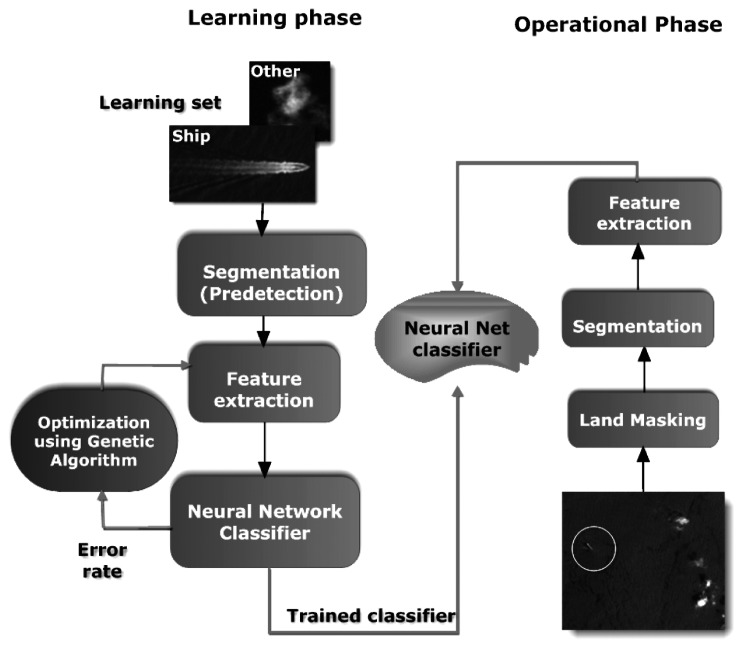
Flow chart of the proposed ship detection algorithm implemented in two phases: a learning phase and an operational phase.

In the third and last step, artificial neural networks are used for the classification of image objects. A neural network architecture is created according to the optimal feature combinations and optimal number of hidden nodes. Figure 1 shows an overview of the approach which consists of two phases: a learning phase and an operational phase.

In the learning phase, GAs are used to train a feed–forward neural network based on reference samples. An objective function is used to calculate fitness that is equal to the inverse of classification error rate. In the operational phase, the best low dimensional neural network architecture is selected as a classifier in the three–step ship detection and classification algorithm.

In what follows, the three main stages of the algorithm are described in detail.

### 2.2. Segmentation (Pre–detection of ship patterns)

Pre–screening of possible ship patterns is based on the contrast between sea (noise–like background) and target (a cluster of bright pixels). The contrast depends on the sea conditions, the ship's detailed shape, and its position relative to the satellite beam. The proposed algorithm applies a 100 x 100 pixel moving window adaptive threshold to the image pixel values (*X_i_*,*_j_*) to discriminate bright pixels [10]. The threshold used for the detection of intensity peaks is based on the mean (*μ_oc_*) and the standard deviation (*σ_oc_*) of the sea background in the moving window.

(1) $$\frac{X_{i,j}-\mu_{oc}}{\sigma_{oc}}\geq \text{Threshold}$$

Noise resulting from image thresholding is removed using a morphological opening operation with a 2 × 2 pixels structural element. Indeed, isolated pixels cannot belong to a ship object, which is usually characterized by a cluster of several bright pixels.

**Figure 2.**
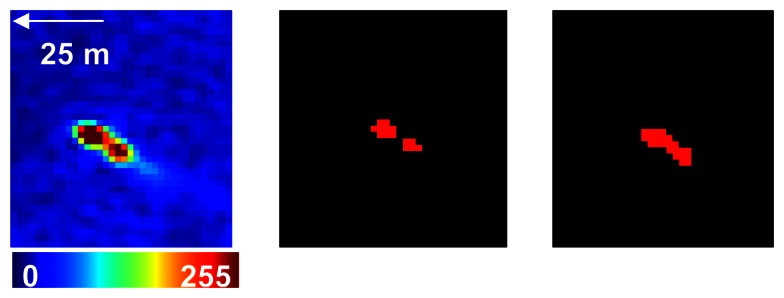
On the left: Panchromatic SPOT–5 (5m) image representing a shrimp boat in pseudo–colours. The middle image shows the two regions of the same boat detected separately during the thresholding operation. The image on the right represents the image object obtained by the region–growing operator.

The resulting thresholded image is then segmented into coherent image objects by means of the region–growing segmentation. Shrimp boats have a characteristic shape, usually consisting of two regions of high intensity related by a region of lower intensity as shown in figure 2. The region–growing operator allows the grouping of the regions of a ship that may be detected separately during the thresholding operation.

### 2.3. Feature extraction

According to [11], image characteristics such as shape and texture are the most useful features in visual interpretation of optical remote sensing images acquired at a high spatial resolution. Notwithstanding their importance, it is difficult to successfully automate the recognition of ships solely based on quantified shape and texture features. Using them in combination with spectral features might result in a better discrimination of ships. Hence, based on *a priori* knowledge of ships' characteristics, we screen out spectral, shape and textural features that most likely characterize ship objects in a unique way, bearing in mind that rotation–position invariance is requisite.

A ship can be generally described by the following characteristics:

– bright pixels,
– large length to width ratio,
– symmetry between its head and tail, like a long narrow ellipse,
– a regular and compact shape,
– ship wakes which have a linear texture.

**Figure 3.**
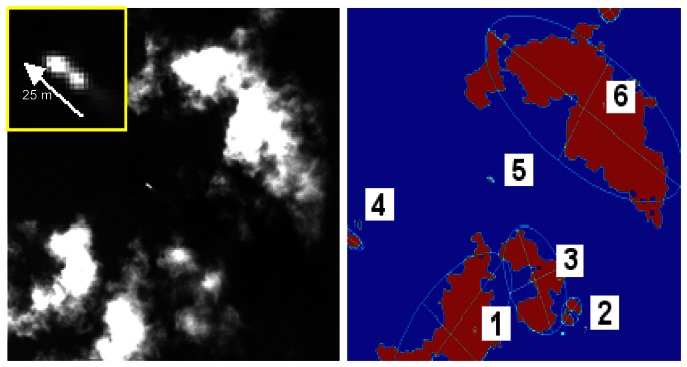
6 image objects obtained by region–growing image segmentation.

Accordingly, 28 spectral, shape and texture features were computed for the image objects. Concerning texture, first and second order texture measures were derived from either the Grey–Level Co–occurrence Matrix (GLCM). Table 1 lists the 28 features calculated for an image object representing a shrimp boat (image object 5, in the example shown on figure 3).

Ship detection can be considered as a 28–dimensional classification problem with two classes: the first class corresponds to ship objects including moving and stationary ships and the second class corresponds to all non–ship objects such as clouds. For classification of such a high–dimensional data set, a large training sample is required. In the case of shrimp boat detection by optical remote sensing, a limited amount of ground truth information is available concerning ship position. The learning performance may not be good in small–sample conditions and with high–dimensional data. For this reason, it is desirable to reduce input dimensionality so as to improve generalization capability and to obtain a network that performs well in terms of both training and test classification accuracies [12]. This underscores the relevance of feature extraction for NNs; e.g., finding the best combination of features in a lower dimensional space that does not lead to a significant decrease in the overall classification accuracy. One way to deal with dimensionality reduction is to use a GA.

A GA is inspired by biological evolution, and is widely believed to be an effective global optimization algorithm. A genetic algorithm consists of a population of genetic strings, referred to as chromosomes, which are evaluated using a fitness function. Chromosomes consist of variables or genes. The fittest chromosomes are then regenerated at the expense of the others. Furthermore, genetic operations such as crossover and mutation are defined. The mutation operator changes individual elements of a chromosome, the crossover operation interchanges parts between strings. The combination of these operations is then repeated during several generations. The intrinsic parallelism of a genetic algorithm, e.g., the ability to manipulate large numbers of chromosomes in parallel, and to handle large, complex, non differentiable and multimodal spaces make the technique a very effective optimization method [13]. The usefulness of GAs in pattern recognition and image processing has been demonstrated [14]. Our approach consists of using a GA to train neural networks by evolving learning parameters and input features [15]. The problem is coded in a binary chromosome with a length of 28 bits (one for each object feature), where the genes have values of 0 or 1. Starting with an initial population of 100 individuals, the selection process selects the healthier ones, directed by the survival–of–the–fittest concept of natural genetic systems. Fitness computation is based on an objective function that is the inverse of classification error rate. For a judgement on their fitness to be made, the individuals have to be decoded to serve as inputs for the NN classifier. The back–propagation algorithm is used for training the network with a learning rate = 0.25 and a momentum = 0.10. A tournament selection procedure and uniform crossover are adopted to select the new population. The probability of crossover is set to 0.5, while the mutation probability is set to 0.05. All the above parameters of the GA are chosen empirically. Within each successive generation, the individuals yielding the highest fitness value, corresponding to the lower classification error rate as measured on a validation set, are enriched in number. The evolutionary process for network refinement is terminated when the number of generations reaches 28. All final offspring individuals thus represent a combination of spectral, shape and texture features that should result in a high classification accuracy. Finally, the best performing low dimensional NN architectures are selected for subsequent classification.

### 2.4. NN image classification

The single hidden layer NN used for the classification is the result of a GA selection procedure employing mutation, different initial weight conditions and uniform crossover. Since it consequently represents the fittest and best performing individuals, its expected error rate is low.

**Figure 4.**
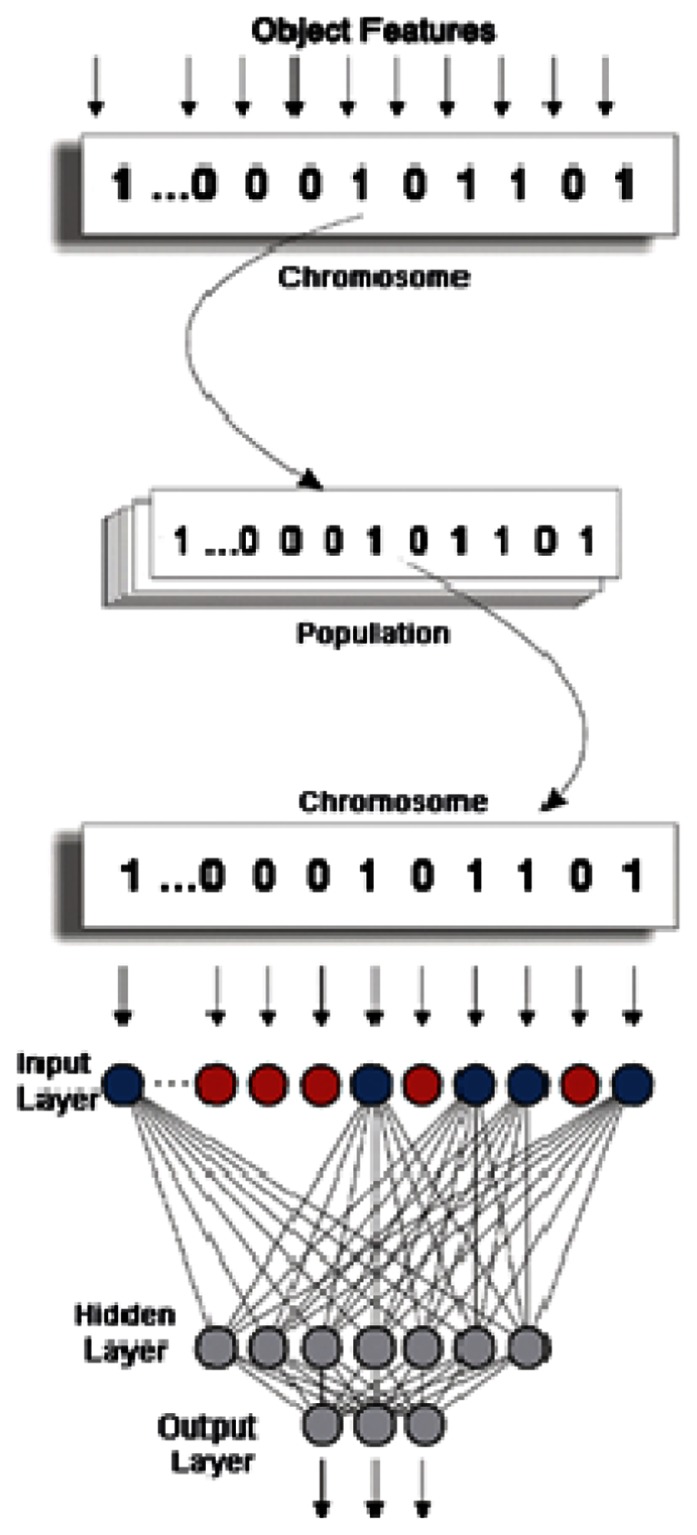
Framework of evolving neural network with a GA (modified from Van Coillie et al., 2007).

Figure 4 illustrates the framework of evolving a three–layered neural network using a GA. In the decoding procedure, all the selected object features valued by 1 are represented by a fully connected input neuron. All other neurons, valued by 0 correspond to non–selected features and are then disconnected. There are two nodes in the output layer, which account for the two classes into which image objects have to be classified. As for the number of hidden nodes in the hidden layer, it is evolved using the GA and follows the rule: number of hidden nodes = number of selected object features/2.

## 3. Method implementation

In this section, we present some experimental results obtained from the application of the proposed algorithm to the detection of shrimp boats. To illustrate the methodology, 7 SPOT–5 images, with a high resolution panchromatic band (5m) were acquired over the Exclusive Economic Zone of French Guiana. The images were provided by the Direct Receiving Station (DRS) of SPOT–5 satellite, operating under the SEAS–Guyane (Survey of Environment of the Amazonia Assisted by Satellites) program. The purpose of this program is to improve flow of image acquisition over the entire Caribbean and Amazonian region. Thanks to SPOT–5's improved resolution and wide imaging swath, which covers 60 x 60 km, the SPOT–5 satellite provides an ideal balance between high resolution and wide–area coverage. The coverage offered by SPOT–5 is a key asset for maritime surveillance in coastal areas and open seas. For the detection of ship targets, panchromatic imagery was preferred over multi–spectral, because additional bytes (bandwidth) of information are better spent on increased resolution than on additional colour.

**Figure 5.**
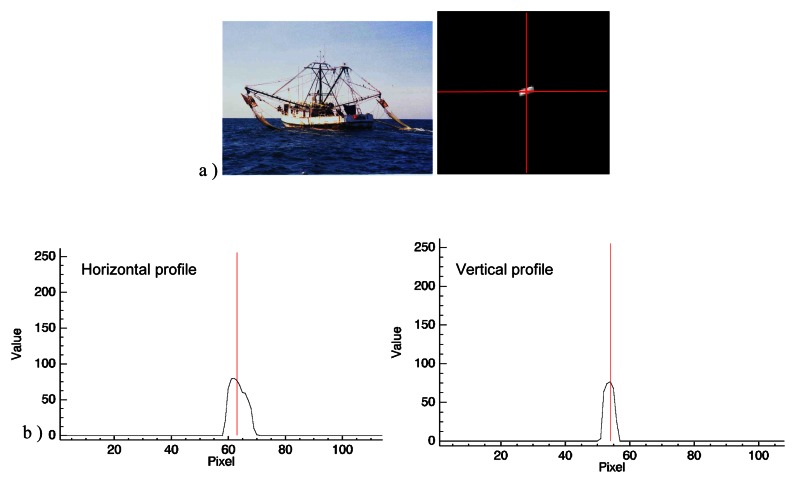
Example of a shrimp boat in the French Guiana area (ranging from 20 to 25 m in length) and its signature on a SPOT–5 image.

On SPOT–5 optical images of 5 m resolution, ships are easy to detect with the human eye, their size is readily estimated and details on the superstructure can easily be discerned. Some of the larger vessel types can be immediately recognised, such as container ships, oil tankers and bulk carriers. Intermediate vessels such as shrimp boats, that range from 20 to 25 m in length still show details, but their interpretation is not so straightforward: it is difficult for an untrained interpreter to discern e.g. a fishing vessel from a patrol boat [16]. Figures 5–a is a typical example of a shrimp boat targeted in this application and the same 25 m long ship imaged by SPOT–5. Figure 5–b shows the pixel intensity values of the shrimp boat across image lines in the horizontal and vertical directions.. As already mentioned, the methodology was applied in two distinct phases: a learning phase and an operational phase.

### 3.1 Learning phase

**Figure 6.**
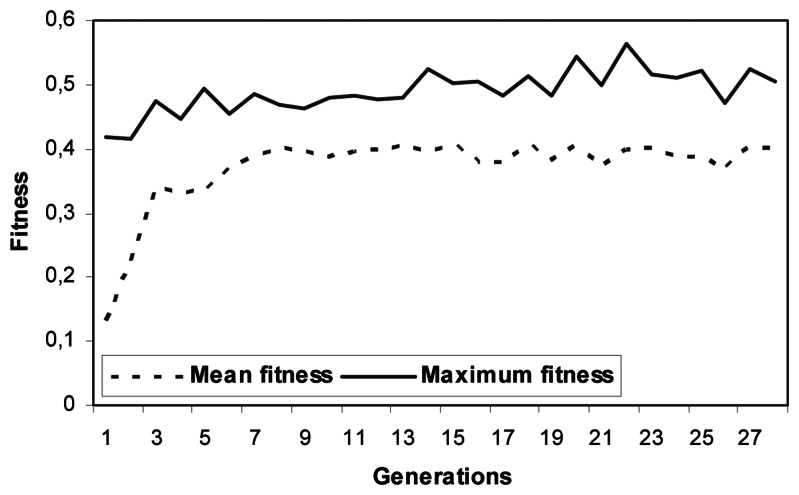
The best fitness corresponding to each generation during the evolutionary training of the NN.

A training set consisting of 200 sample objects, among which 61 represent shrimp boats, was used during the learning phase. This training set was obtained from 5 SPOT–5 images. The two remaining images, acquired on 13 August 2003 and 03 July 2007, were used for the evaluation of the algorithm's performance. The network was trained for up to 4000 epochs with the GA. The output of the optimization procedure is represented in figure 6.

On this figure, we can see the variation of best fitness represented by the best chromosome with the number of generations of the GA. We notice that the best chromosome is located at the 22^nd^ generation with a maximum fitness of 0.56.

Hence we can determine which features played a significant role for the classification, and what features were useless for, or even disturbed, the NN classifier. Among the 28 initial features calculated for the image objects, only 8 features were extracted: Number of pixels, Mean, Standard deviation, Minimum, Maximum, Variance, Ratio Major/Minor and Texture uniformity. Hence, the optimal NN architecture consisted of 4 nodes in the hidden layer as determined by the GA. An average value of 0.1377 (95% confidence interval: 0.0854; 0.1899) was obtained for the generalization error of the optimal NN, estimated by means of 10–fold cross–validation.

The effect of the GA–driven feature selection on the detection of shrimp boats was evaluated in the operational phase.

### 3.2 Operational phase

**Figure 7.**
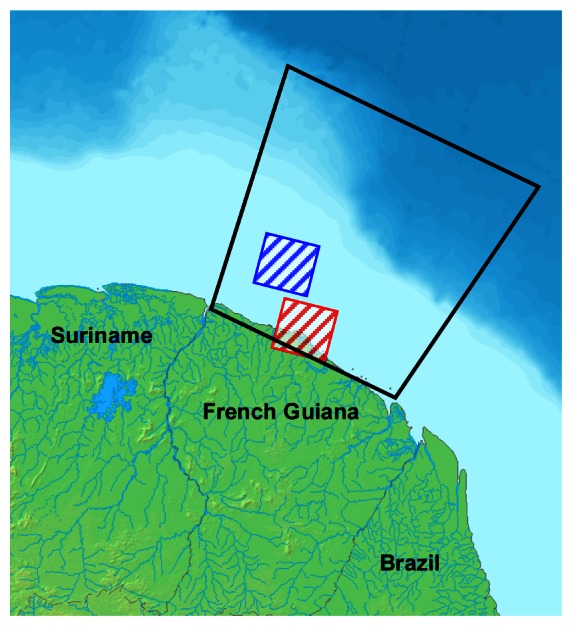
Spatial coverage of the SPOT–5 images acquired for the validation of the algorithm: the red and blue hatched squares correspond to the images acquired respectively on 13 August 2003 and on 03 July 2007. The black square represents the spatial extent of the EEZ.

Two images acquired in extremely contrasting meteorological conditions were used to evaluate the algorithm's performance in the operational phase (figure 7). This allows to roughly explore the domain of validity the algorithm. The image acquired on 13 August 2003 is characterized by an almost cloud–free sea surface and a low sea state. Conversely the image acquired on 03 July 2007 represents very unfavourable situations of high percentages of cloud cover and high sea state.

When applying the algorithm for the entire scene acquired on 13 August 2003, a land mask is needed so not to mistake land for ship objects. A global coastline database with a high accuracy was therefore a necessary element of the operational system. Once the land mask was applied, the image was submitted to the three processing stages that constitute the proposed algorithm. In a network population of 100 individuals, 8 fully connected neurons resulting from feature extraction using the GA were used for the classification.

**Figure 8.**
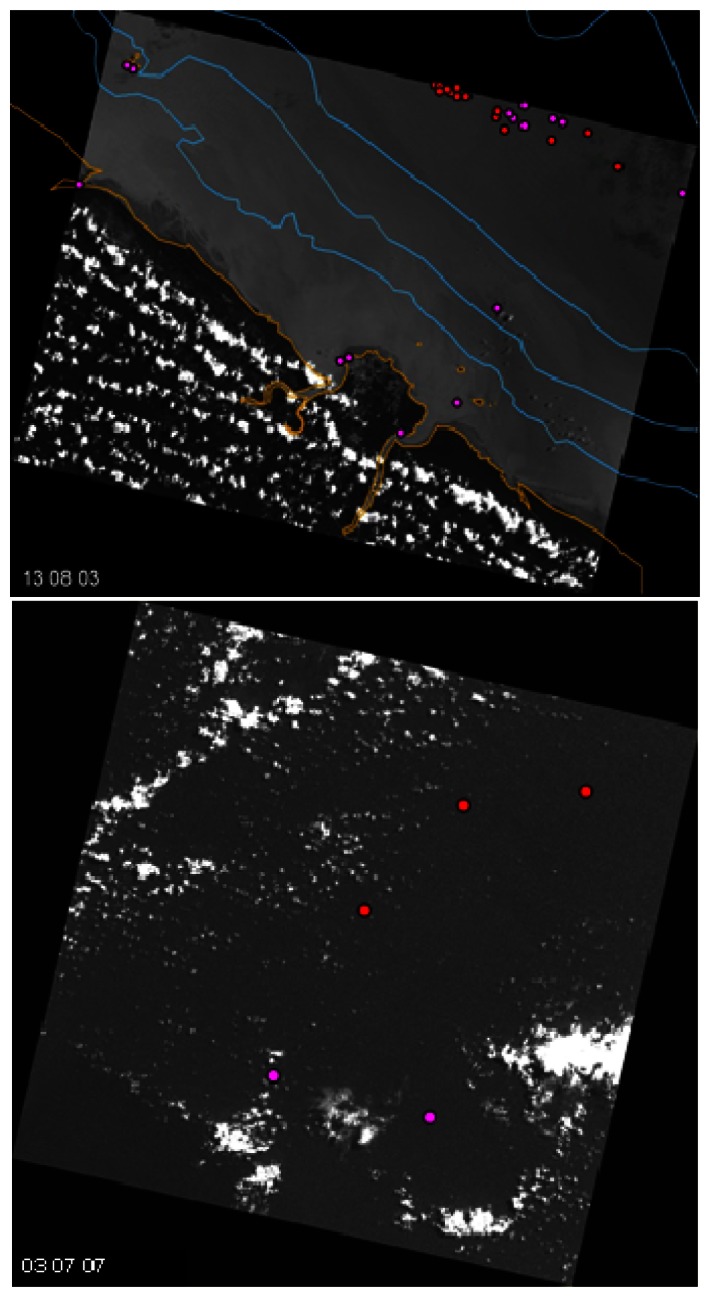
Automatically detected ships on August 2003 image (a) and July 2007 image (b) are represented by red circles. Pink circles correspond to non–detected ships.

The results for shrimp boat detection on the two SPOT–5 images using the developed algorithm are represented in figures 8–a and 8–b. It is generally difficult to correctly cross–check the results of automatic ship detection because only limited ground truth information is available concerning ship positions. Moreover, unavailability of Automatic Identification System (AIS) data in French Guiana precluded a correct validation of the algorithm's performance. Nevertheless, in our case, visual interpretation by trained human operators was used to help assess performance.

Performance was measured by detection rate (DR) and false alarm rate (FAR). DR is the number of shrimp boats correctly detected as a percentage of the total number of real shrimp boats and FAR is the number of shrimp boats incorrectly reported as a percentage of total number of real shrimp boats. On August image (Figure 8–a), it was found that the 16 shrimp boats detected by the system perfectly matched the operator reported ships' positions. Hence, FAR for class‘ship’ was equal to 0. According to the operators' report, a total of 31 ships were identified in the entire scene. Among them, 10 ships were less than 14 m long, and there was one moored ship. This means that there were possibly 20 real shrimp boats. Detection rate as referenced in this way to the 20 possible shrimp boats was thus found to be equal to 80%, which is quite high. A much lower detection rate of only 52% was reported when taking as a reference the total number of boats identified by the human operator.

Compared with the algorithm's performance on the cloud–free image, the results on the July image (Figure 8–b), showed a marked underperformance. The detection rate was found to be equal to 60% with a corresponding false alarm rate of 5700 %. By adjusting the threshold during the pre–processing stage (equation 1 on section 2.2), the false alarm rate could be reduced but only at the cost of a decrease in the detection rate.

The output of the system is a list of detected ships' positions and ancillary information related to the ships' lengths and headings (for moving targets). In its current state, the system does not allow speed extraction; instead, the detected ships are categorized into two categories: ‘in motion’ or ‘static’.

Of particular interest are those detected shrimp boats for which no corresponding VMS is reported, highlighting potential unreported fishing activity. Close inspection of these targets by patrol aircraft, for example, may be required in some cases. Consequently time delay becomes a requirement for an operational system for the automatic detection of ships ndash; the total delay should be below 1 hour to allow for meaningful follow–up action. Therefore another aspect taken into consideration in performance evaluation was the timeliness. When applied on an entire SPOT–5 image, the system allowed delivery of end results within 1 hour from image acquisition, thus proving its suitability for near real–time monitoring of fishing activities.

## 4. Discussion

The implementation of the proposed algorithm on a 5–m resolution panchromatic image showed that, in favourable conditions, as in the case of the August image, the system can provide reliable detection of shrimp boats with minimal operator intervention and practically without any false alarms. Failure to detect targets of less than 14 m length may be explained by the insufficient spatial resolution of 5 m used for the detection of small ships. A spatial resolution of better than 3 m might be required in order to provide a better detection rate. For most of the detected ships, the algorithm overestimated ships' lengths compared to visually extracted sizes. This is a result of particular image conditions: almost all the ships were in motion. On optical imagery, the moving ship and its near wake are difficult to separate because they are connected and can have similar brightness, so wake and (moving) ship detection often amount to the same. Therefore during segmentation, a ship and its wake were considered as being part of the same image object, resulting in an overestimation of ship size.

In very unfavourable situations of special types of background clutter arising in particular meteorological situations and high percentage of cloud cover, as in the case of the July image, the performance degrades due to extremely high false alarm rates. In these situations, the fully automatic results may still be improved by human corrections, such as manually adapting the detection threshold or visually discarding the false alarms. However it is generally assumed that such situations may be avoided in operational circumstances.

Even though the overall results are promising, there still remain several issues that need improvement or refining in order to render ship detection from optical satellite imagery fully operational:

– It is acknowledged that the opportunity of the artificial NN to learn class appearance is influenced by the composition and the size of the training set. In this study, the small size of the available training set, due to practical limits, had a significant effect on the performance of the algorithm. Further tests on additional representative samples are currently underway.
– To further improve our quantitative knowledge of detection capabilities, more work needs to be done into testing the algorithms' performance under different weathering conditions. Depending on the amount of false alarms that would be obtained following these tests, it would be possible to integrate weather and oceanographic data to reduce false alarms rates.
– The present work needs to be extended to implement an automatic wake detection approach so as to improve ship length classification and speed estimation.
– A great deal of effort is currently being undertaken to improve validation procedures and control efficiency by i) introducing information from other maritime monitoring systems, such as VMS, ii) cross–cuing to other sensors, such as the Synthetic Aperture Radar (SAR) sensor, for obtaining or confirming classification.

## 5. Conclusion

This paper has demonstrated the following:

– the feasibility of ship pattern recognition from high spatial resolution satellite imagery,
– the appropriateness of a feature–based approach for ship detection and
– the viability of utilizing neural networks evolved by GAs in classifying shrimp boats.

From an application point of view, the most remarkable benefit is the great contribution to the detection of illegal fishing activities, especially in areas where AIS information is unavailable. Additionally, the modest requirements in terms of computer and hardware of the system offer a potential for providing a recognition operational system to a variety of users such as coast guards, search and rescue organizations and harbor masters. If these entities have access to optical space borne data they could complement SAR and ground based monitoring using the advantages of the developed prototype, mainly:

– possibly better determination of ship position,
– the possibility of using manual interpretation for a refinement of ship's classification.

Further work on advanced ship detection techniques is still warranted. Concerning the work presented in this paper, future research will include tests with other datasets for various ships and environmental conditions; study of sea state effects, antenna gain and ship motion on detection performance; and evaluation of the algorithm using very high spatial resolution optical imagery (sub–metric pixel resolution).

## Figures and Tables

**Table 1.** List of spectral, shape and texture features calculated for image objects. An example of feature values is provided for image object 5 of figure 3 (*M = momentum of inertia, *GLCM= Gray Level Co–occurrence Matrix).

**Spectral**	Number of pixels	39
	Mean	189,41
	Standard deviation	57,05
	Sum of squares	1,52E+06
	Min	95
	Max	255
	Variation	3254,30
	Asymmetry coefficient	0,49
	Kurtosis	0,42
**Shape**	Perimeter	23,90
	Area	39,00
	Compactness	0,86
	Elongation	2,44
	M1 *	0,23
	M2	0,03
	M3	6,59E+14
	M4	7,33E+13
	Major axe	11,14
	Minor axe	4,54
	Ratio Major/Minor	2,46
	Eccentricity	357,84
**Texture**	GLCM* mean	150,00
	GLCM variance	1,08E+06
	GLCM uniformity	404,00
	GLCM inertia	4,97E+05
	GLCM correlation	5,01E+05
	GLCM entropy	99,55
	GLCM homogeneity	22,00
